# The Critical Factors Affecting the Deployment and Scaling of Healthcare AI: Viewpoint from an Experienced Medical Center

**DOI:** 10.3390/healthcare9060685

**Published:** 2021-06-07

**Authors:** Chung-Feng Liu, Chien-Cheng Huang, Jhi-Joung Wang, Kuang-Ming Kuo, Chia-Jung Chen

**Affiliations:** 1 Department of Medical Research, Chi Mei Medical Center, Tainan 71004, Taiwan; chungfengliu@gmail.com; 2 Department of Emergency Medicine, Chi Mei Medical Center, Tainan 71004, Taiwan; jasonhuang0803@gmail.com; 3 Department of Environmental and Occupational Health, College of Medicine, National Cheng Kung University, Tainan 701401, Taiwan; 4 Department of Anesthesiology, Chi Mei Medical Center, Tainan 71004, Taiwan; 400002@mail.chimei.org.tw; 5 Department of Anesthesiology, National Defense Medical Center, Taipei 11490, Taiwan; 6 Department of Business Management, National United University, Miaoli 36003, Taiwan; kmkuo@nuu.edu.tw; 7 Department of Information Systems, Chi Mei Medical Center, Tainan 71004, Taiwan

**Keywords:** artificial intelligence, healthcare AI, deployment and scaling, medical center, critical factors, stakeholders

## Abstract

Healthcare Artificial Intelligence (AI) has the greatest opportunity for development. Since healthcare and technology are two of Taiwan’s most competitive industries, the development of healthcare AI is an excellent chance for Taiwan to improve its health-related services. From the perspective of economic development, promoting healthcare AI must be a top priority. However, despite having many breakthroughs in research and pilot projects, healthcare AI is still considered rare and is broadly used in the healthcare setting. Based on a medical center in Taiwan that has introduced a variety of healthcare AI into practice, this study discussed and analyzed the issues and concerns in the development and scaling of medical AIs from the perspective of various stakeholders in the healthcare setting, including the government, healthcare institutions, users (healthcare workers), and AI providers. The present study also identified critical influential factors for the deployment and scaling of healthcare AI. It is hoped that this paper can serve as an important reference for the advancement of healthcare AI not only in Taiwan but also in other countries.

## 1. Introduction

AI has been given a lot of attention worldwide due to its continued significant scientific and technological advancements. People from all walks of life have gained tremendous interest in AI-related technologies resulting in the development of several amazing AI-related products. For example, the manufacturing industry is now utilizing intelligent solutions through AI in many of its areas, such as defect identification on the production line, automatic process control, predictive maintenance, and raw mix optimization (e.g., [1]). Some industries are using AI for the development of unmanned factories. AI-related research has grown substantially in the past 10 years [2], but most of them only reported the quality of prediction models (such as accuracy, sensitivity, specificity, and Area Under Curve (AUC) values), which makes it difficult to judge their practical feasibility. In terms of the use of AI in healthcare, there is still much space for enhancement, which could overturn the ecology of traditional healthcare. According to Gartner’s report [3], 54% of data science projects in most organizations were never deployed or only partially deployed in practice. Indeed, different from other industries, the healthcare industry is strictly limited and restricted by many laws and regulations. Therefore, the experience of using AI in general industries cannot be directly applied to the healthcare industry (an unmanned hospital is obviously impossible).

Two of Taiwan’s most competitive industries are healthcare and technology. The development of healthcare AI is an excellent opportunity for Taiwan since it is becoming increasingly popular and could potentially help a lot of people. Therefore, from the perspective of economic development, it is urgent to promote healthcare AI. Two of the four AI Research Centers established in 2018 by the Ministry of Science and Technology in Taiwan are connected with the healthcare industry (one is located at the National Taiwan University and the other at the National Cheng Kung University). The establishment of these centers allows the Taiwanese government to improve the overall quality of the nation’s healthcare through AI. The development of healthcare AI also aims to lower the total medical expenditures at a national level. Moreover, as the positive benefits of AI are gradually being recognized and emphasized by healthcare institutions, relevant specialized units have sprung up (e.g., Big data center and AI center) to perform more research and development. Healthcare AI is considered as one of the industries where AI has the most opportunities for development [4,5]. Therefore, many technological manufacturers and start-ups invest heavily in innovative development, hoping to gain an advantage and to initiate the trend in healthcare AI. However, it is important to determine how the biggest consumers of healthcare AI, namely the hospitals and healthcare workers, will react towards such a trend. Whether the hospital obtained AI by developing it or through external purchase, it would require a vast amount of time, special manpower, including AI engineers, and financial resources for building basic infrastructures (GPU servers, data warehouse, and development platform), purchasing various AI products, and storing and transforming big-data. Therefore, calculating the obtainable investment efficiency is the most fundamental and significant concern for hospitals in AI evaluation and development.

The healthcare industry can simplify the calculation of investment efficiency of AI by measuring the income increase, cost reduction, and quality improvement. After this, they could assess the working efficiency improvement, working pressure reduction, and patient satisfaction improvement. In Taiwan, hospital income mainly comes from the national health insurance (NHI) payment under the global budget payment system, which is not directly correlated to the type of equipment and devices used in hospitals (AI could be viewed as a kind of equipment). This means that the introduction of AI in hospitals may not directly generate new sources of income. Additionally, many scholars pointed out that AI performs better than other medical professionals, such as in medical image interpretation (e.g., [6]). Furthermore, many have begun to question whether healthcare workers could be replaced by AI (e.g., [7]). As a response, the government proposed clear regulations to stipulate how the number of healthcare workers should match with the hospital scale. Hence, it may be impractical to say that AI could reduce the volume of healthcare workers in the short term. Additionally, there may be limitations in using AI to reduce the deployment of equipment and devices, and the acceptance of AI by healthcare workers also deserves attention. Since hospitals tend to be busy, many healthcare workers believe that using AI will only consume their time and attention, which they could use in attending to patients instead. Moreover, they believe that they can accurately evaluate some disease outcome changes even without AI; thus, it is safe to assume that some healthcare workers lack the willingness to use AI.

The authors of this paper are affiliated with Chi Mei Hospital, a hospital with three branches in Taiwan. In August 2019, Chi Mei Hospital established the AI Center in its general faculty as the base for developing healthcare AI. Relying on big data accumulated over ten years, the hospital has focused on developing AI-smart clinical outcome prediction. As of March 2021, it has self-developed 15 kinds of AI systems, which are then coordinated with the existing Hospital Information System (HIS) to help healthcare workers in their clinical decision. So far, all of the hospital’s branches have installed the AI systems in several departments, including the Emergency, Surgery, Anesthesiology, Intensive Care Unit, and Nursing departments. In addition, as the AI Center is gradually developing and improving the AI systems, it was able to study and observe the use of AI in the healthcare setting and publish them in international journals [8,9]. Based on the experience of Chi Mei Hospital in the implementation of its AI systems, this paper reported the current situation and the challenges being faced by healthcare AI from the perspectives of the government, hospitals, users, and manufacturers. It also determined the key factors affecting the deployment of healthcare AI.

## 2. Development of AI in Chi Mei Hospital

### 2.1. AI Computing Infrastructure

**Figure 1:**
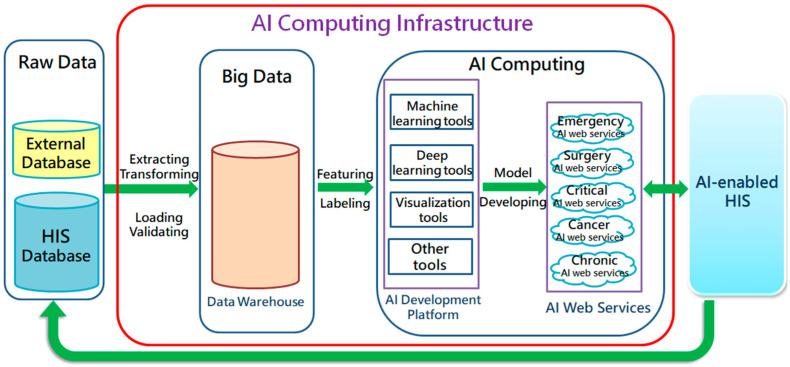
AI Computing Infrastructure of Chi Mei Hospital.

Supported by the Board of directors and the Superintendent, Chi Mei Medical Center built the Center for Big Medical Data and AI Computing (hereinafter referred to as AI center) in May 2019. As the base for developing AI in the three branches of the hospital, this center has two main tasks: (1) to establish Big Medical Data (data warehouse) and (2) to develop AI applications. It is hoped that AI will not only be used for academic research but is also expected to produce practical applications for clinical use. The Big Medical Data acts as a retrospective data source for AI development and healthcare researches, and its main source is the online database of HIS. Since the structure of the data warehouse and HIS database are very different from each other, it makes this project enormous and complicated. Therefore, the AI Center usually develops and establishes the topic-specific big database with the most researchers first. To do this, the Department of Information Systems (IS) in hospitals is tasked to help in transferring the needed data from the HIS database. Figure 1 shows the AI Computing Infrastructure of Chi Mei Hospital.

* HIS: Hospital Information System; AI: Artificial Intelligence. 

**Figure 2:**
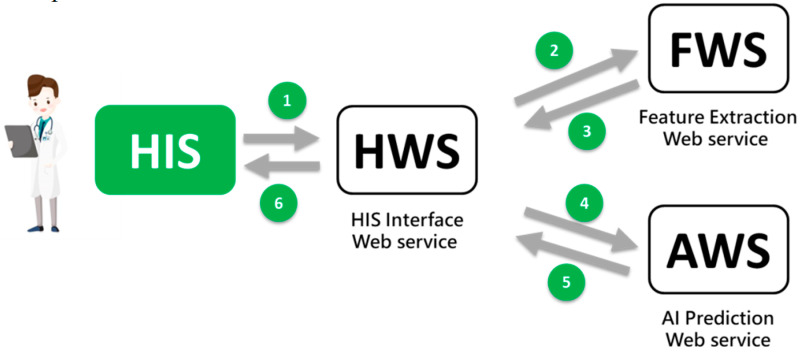
AI Web Service Interaction.

Based on the Service-oriented Architecture (SOA), the IS department designed three types of web service program (WS) that interacts with HIS to process the AI prediction (Figure 2). These are as follows:

- HIS interface WS (HWS)
  The HWS receives calls from the existing (HIS) and sends the prediction result (e.g., risk probability) back to the HIS.
- Feature extraction WS (FWS)
  The FWS receives calls from HWS, retrieves the patient’s characteristic values (such as age, blood pressure, lab data, etc.), and sends them back to HWS. This may include the use of IoT technology to retrieve physiological information at the bedside.
- AI prediction WS (AWS)
  The AWS receives calls from HWS, enters the acquired feature values of the patient, performs the AI prediction, and returns the result to HWS.

Six sending/receiving messages are completed while a prediction is triggered by users (healthcare workers). They contain only short messages and only cause minor impacts on the online HIS. So far, 15 healthcare AI predictions (15 AI WSs) are being used on the AI Web Services server (see Figure 1), which is routinely monitored by IS engineers. Because AI is positioned as an assistance role, system failure has little effect on the overall clinical operation.

* HIS: Hospital Information System; AI: Artificial Intelligence

### 2.2. Promotion Strategy

**Figure 3:**
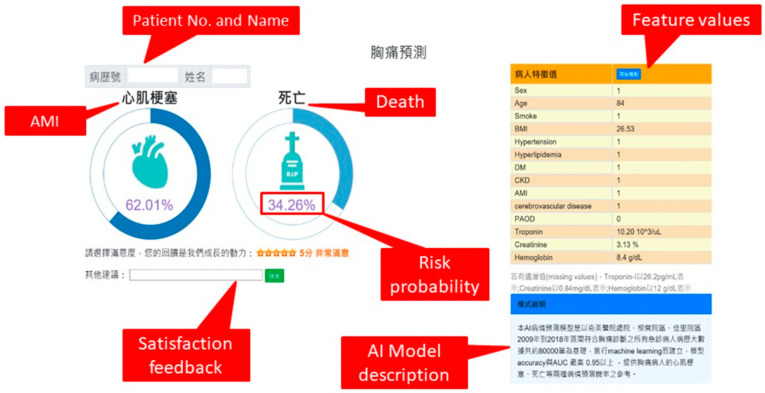
A Screenshot of a Deployed AI System.

To demonstrate its commitment to developing AI, Chi Mei Hospital has subsidized the training of hundreds of healthcare workers in AI practice. Each training lasts for 4 weeks to 4 months, depending on the complexity of the AI. Moreover, the AI Center has assigned different personnel to give mini-lectures on how to develop AI and create specialized individual roles, evoking wide repercussions among healthcare workers. Because of this, the hospital’s workers no longer feel that AI is inaccessible, allowing them to propose various ideas proactively. To determine the plausibility of the staff’s ideas, inter-disciplinary meetings with healthcare workers, AI analysts, and IT engineers as participants are being held. The approved ideas are then constructed as AI projects and finally implemented and used in practice. Since the establishment of the AI Center, the emergency department (ED) was the first department to join the AI development and has completed a variety of disease outcome prediction systems (e.g., older patients with influenza, patients with chest pain). Due to the success of the AI systems implementation in the ED, the AI center revised the systems depending on the needs of the other departments and promoted their use (e.g., outcome prediction of burns for surgical treatment, anesthesia risk assessment, mortality prediction and timing prediction for weaning mechanical ventilation in ICU, and fall detection in elderly wards). Figure 3 shows a screenshot of the outcome prediction system in ED patients with chest pain [8]. The system has been integrated with the existing emergency computerized order entry system. Until 30 April 2021, a total of 50 AI projects have been proposed mainly focusing on physicians’ clinical requirements; 15 of which have been deployed in the clinical practice (integrated into the existing HIS); 25 are still being completed; 10 have been completed but have not yet been implemented. Among the 10 completed projects, five have not yet been used because the physicians considered their model quality not good enough for clinical use (model AUC < 0.7) and need improvement, while the other five projects are scheduled for further development but are under slow progress due to heavy workload in the IS department.

* AMI: Acute Myocardial Infarction

Because of previous promotions, the heads of each hospital department are aware of the benefits of AI in healthcare; thus, each gives enough time to discuss AI issues and solve them based on consensus through regular department meetings. Moreover, each department designates specific groups that target specific clinical demands to create AI projects to be submitted to the AI Center. After confirmation and revision, the department can apply the proposed project into practice. In this way, departments are able to carry out subsequent launches of AI systems smoothly and reduce resistance from other healthcare workers. To promote AI, Chi Mei considers it as an assistive tool rather than a substitute for humans’ skills and intellect. The hospital also believes that healthcare workers have the right to choose whether they will or will not use AI and shall not suffer any punishment or salary deduction for not using it. Various departments welcomed such policies, which brought out more projects for practical application.

Whether preparing big data or integrating models with HIS, the support of the information department is very much critical. However, the daily workload of the IS department is quite heavy, and understandably, it cannot provide much support on AI development. Therefore, Chi Mei has allotted a data-processing fee in the budget of the in-hospital projects of AI Development every year to encourage IS engineers to support AI development in their free time (off-work time), which has been a feasible and effective approach.

### 2.3. Emerging Benefits of AI Adoption

AI benefit evaluation is critical but not easy to measure. However, the benefits of Chi Mei’s deployment and scaling of AI have gradually emerged. For example, on the basis of no significant differences in gender, age, and disease severity among patients, the periods before and after adopting AI timing prediction for weaning mechanical ventilation in ICU (2019/7–11 vs. 2020/7–11) were compared. It was found that the average time of using a ventilator was reduced by about 22 h while having the same medical quality. This proves that not all the benefits of using AI applications are obvious and may not be recognized in a single AI factor. Therefore, it requires continuous in-depth observation and evaluation.

## 3. Viewpoint of Stakeholders

The most fundamental consideration for every transaction is the cost-benefit, even for the purchase of information communication technology (ICT) devices. Therefore, the first step to promote healthcare AI is to have careful considerations of the benefits that consumers could obtain by adopting it. These consumers are the most important stakeholders in the development of AI, comprising of the government, healthcare institutions (hospitals), end-users (healthcare workers), and AI providers. Based on the hospital’s experience, literature review, and actual observations, this study discussed the viewpoint of AI stakeholders.

### 3.1. The Government

Using smart technologies to improve healthcare quality has long been one of the government’s scientific and technological policies. In the National Healthcare Quality Award (NHQA), an annual competition held by the Joint Commission of Taiwan for hospital accreditation, smart healthcare is always an item of focus. Every year nearly 200 groups attend this competition, and most are from hospitals. Moreover, the truth is, the overall healthcare spending and healthcare insurance expenditure has become increasingly unbalanced. Since technologies could improve public health by both early prevention and intermediary and tertiary care, hospitals could apply AI to different healthcare services on the premise of accurate trend prediction and risk of individual disease outcome change. Thus, through AI, the government could reduce the previously undifferentiated healthcare policies (e.g., nationwide disease screening) and further control the overall healthcare expenditure with guaranteed healthcare quality. After all, national health insurance and healthcare occupy large proportions of the national budget. If AI technologies could accurately predict the public healthcare trend and the epidemiological pattern of diseases and help hospitals plan corresponding strategies with precision, it may create concrete benefits by promoting the government’s healthcare and welfare policies. Additionally, from the perspective of detail-oriented healthcare resource management, AI could help avoid healthcare wastes (such as lower examination and medication), meeting the expectations of the government towards smart technology. Therefore, in the short term, the government should be committed to formulating and revising regulations, such as insurance reimbursement policy or specific subsidy programs, to encourage the development and adoption of AI in healthcare institutions.

Another important role of the government is as an industrial promoter. The Taiwan government may pledge AI funding schemes, such as the strategies used in the US and Germany [10,11], for promoting joint efforts in creating breakthroughs in AI. As Taiwan embraces complete big data in national health insurance, it is essential for the government to formulate regulations of data governance [12] and release authorization for the development of the AI industry.

### 3.2. Healthcare Institutions

Different from other industries, healthcare institutions (e.g., hospitals) are under the strict supervision of various policies and rules regarding their environment, workforce, and medical device deployment. For instance, in Taiwan, the Establishment Standards for Medical Institutions has stipulated the human resource arrangement in hospitals providing minimum requirements on the type and the number of medical professionals in a hospital (to be more specific, a minimum of two physicians for every 10 beds; a minimum of two nurses for every three beds for hospitals over 50 beds; a minimum of one pharmacist for every 40 beds; a minimum of three clinical laboratory technologist for every 50 beds; a minimum of two radiologists for every 35 beds). Even though the introduction of AI into healthcare institutions may bring out promising benefits (e.g., the AI-assisted medical image interpretation), it cannot significantly reduce the actual cost of manpower due to the restrictions of laws and regulations. In fact, the scope of payment under the NHI system is based on the total amount and the Diagnosis Related Groups (DRG), aside from calculating the relative declarations of each healthcare institution and reasonable load for outpatient services in recent years (referring to the Standard Reimbursements for Medical Services and Treatment for the National Health Insurance in Taiwan). As a result, hospitals can barely accumulate surpluses from the NHI system, and some treatments may even operate at a loss, which AI’s introduction cannot simply change.

Compared with the control of manpower allocation, the government has relatively no explicit regulations on medical equipment. However, the “arms race” is used as a major promotion topic for the healthcare industry, at least in Taiwan. As can be seen from the popularity of the costly “da Vinci Surgical System” in large hospitals in Taiwan, those hospitals are not reserved in investing in medical equipment. The added value of AI in medical equipment (e.g., the ventilator may be equipped with AI patient risk prediction) could increase the attractiveness of the equipment. AI providers have proposed many innovative diagnostic methods, but hospitals need to confirm first whether these could replace the original ones, especially in terms of practical use. For example, AI could help identify the risk of Obstructive Sleep Apnea Syndrome (OSAS) based on patients’ neck CT scans. However, healthcare professionals need to rigorously determine if they could use AI as a reference for diagnosis (traditional diagnosis of OSAS includes nocturnal polysomnography).

The manufacturing industry can establish unmanned factories thanks to smart technologies, but hospitals are different; they cannot run without medical professionals. After the introduction of smart technologies, the manufacturing industry can easily calculate quantitative benefits by measuring production increase, manpower reduction, and yield (healthcare quality) improvement. However, it is difficult for hospitals to reflect the same quantitative benefits because there is lesser variation in production capacity (e.g., the outpatient visit is regulated by Reasonable Load for Outpatient Services Policy), manpower (the healthcare staffing quota is limited by the Establishment Standards for Medical Institutions), and yield (the healthcare quality is in line with hospital accreditation standard). Hence, hospital operators are suspicious about the necessity to invest huge capital in AI development. At present, most hospitals initially use AI for education and research and not for clinical purposes because they believe that AI can be helpful in academic research (research publication is required in teaching hospitals). As for the clinical benefits of AI, hospitals need more time to observe and assess the results.

Other organizational and managerial challenges such as organizational resistance to data sharing and lack of strategy for AI development were pointed out in previous research [13], which could also appear in healthcare institutions and need to be overcome as well.

### 3.3. End Users (Healthcare Workers)

Based on the user-centered perspectives, understanding the clinical needs of healthcare workers and the difficulties they face in clinical decision-making is the basic principle for the development of healthcare AI. The “coolness” of technology should not be given too much attention as it may generate unnecessary AI, which could neglect the real purpose of AI development, that is, to improve clinical practice.

Furthermore, the cultivation of healthcare workers requires rigorous education and high cost. If technology such as AI could partly replace manpower, it would be called an epoch-making healthcare revolution. However, medical education based on evidence usually emphasizes the accumulation of clinical experience and related skills aside from formal school education. Moreover, practical training is highly significant in medical professionals’ practice; this is why many professional units hold regular discussions for clinical cases. In addition, the Objective Structured Clinical Examination (OSCE) provides strict testing of professional skills for healthcare workers. If healthcare professionals rely too much on AI’s assistance, they may not develop appropriate professional skills and experience. Therefore, although Chi Mei has introduced AI medical image interpretation, they have discouraged (and even prohibited) medical interns or resident physicians from using it. Nevertheless, the influential factors for medical decisions may be too many; using AI as a tool to assist decision-making and provide an additional layer of gatekeepers may reduce the chance of negligence or even misjudgment, and AI will have its value. However, there is no denying that in the long run, healthcare workers may worry about being replaced by AI, and this still requires careful attention [13].

The black-box problem of AI is another major reason that affects healthcare workers’ acceptance of AI. Even though AI is highly accurate, healthcare workers cannot completely trust its suggestions if the reference or logic is unclear. After all, life is above all, and healthcare workers would be to blame if anything untoward happens. Hence, it is very important to guarantee and improve healthcare AI’s explainability [14,15].

Since hospitals are always busy and healthcare workers are under great pressure, hospital management should introduce AI gradually without increasing the complexity of the care process. To achieve this goal, the AI functions should be integrated seamlessly with the existing HIS or operate automatically as much as possible (while retaining the final decision to the workers) with utmost convenience. Finally, instead of forcing healthcare workers to follow, they should have the right to choose whether they will use AI’s suggestions. As long as the AI can perform well consistently in a long time, healthcare workers would gradually accept and routinely use it.

### 3.4. AI Providers

“To create an AI physician” is the most dreamlike goal of AI development in health care. Many technology providers such as IBM, Google, Microsoft, and other start-ups have all invested in healthcare AI, creating highly innovative products. However, as the profits seem to be unapparent, many AI providers keep losing money, which prompted them to reduce their investment. The sharp cut-down of personnel in the IBM Watson Health Department is an obvious example [16]. As mentioned previously, the medical industry is very different from other industries, and its development and operation are subject to many strict regulations. It may be far from reality to replace healthcare workers with AI. In other words, the development of healthcare AI shall not follow the same way as that of general industries. According to surveys, even though AI and real physicians have the same diagnostic quality, the public would still prefer real physicians. Moreover, even if the AI provides the judgment result, the patient hopes that a real physician can make the final confirmation [17].

The choice of the user-centered target [18] is key in determining the success or failure of AI. If AI products are innovative but difficult or non-critical to use in the hospitals, the investment of providers will just go to waste. Thus, the providers must design AI products based on actual demands or requirements of the healthcare industry rather than technological innovation. AI providers should assign someone (preferably a healthcare professional) who will be tasked to comprehensively understand the needs of the medical field and propose convenient and flexible AI solutions. Particularly, in recent years, several studies proposed precision and personalized medicine that emphasized the complexity of medical factors requiring personalized care and not a “one-size-fits-all” protocol [19,20]. For instance, although the diagnosis of malignant lesions through X-ray may be the same all over the world, the outcome prediction and treatment may vary with the nationality, race, sex, age, eating habits, and social status of the patient. Therefore, before development, providers must determine whether the AI product is for general or special use. Aside from this, AI development requires thorough considerations in terms of user number, obvious effectiveness, and explainability of results.

High-quality big data is an important prerequisite for AI development, which usually comes from the cooperation between healthcare institutions and providers (developers) rather than from providers alone. Therefore, providers need to select qualified healthcare institutions for long-term cooperation and carefully store big data. Additionally, to avoid interference in healthcare operations, AI products should provide convenient interfaces to combine with the existing HIS of healthcare institutions. Further, an AI with a total solution is more competitive than a single function AI. For example, for patients diagnosed with diabetes mellitus, AI should suggest an ophthalmoscopy test and extract images by IoT. Next, it should evaluate the risk for diabetic retinopathy and provide suggestions (combined with other laboratory data of patients) to confirm the diagnosis (could be sent to mobile phones of attending physicians). Moreover, an AI that integrates Business Intelligence (BI, e.g., digital dashboard), Internet of things (IoT), wearable devices, mobile, and remote technologies in the early and late stages of health care would be great added features.

It is an interesting issue to explore whether AI plays a “leading role” or “supporting role” in the healthcare industry; that is, it is essential to determine whether it is worthwhile to create AI products to attract investments from healthcare institutions. For instance, it would be good to know if hospitals would prefer to purchase an X-ray AI interpretation system alone and integrate it with their existing X-ray machines to assist radiologists or purchase built-in AI interpretation X-ray machines at a higher price. According to our observations, AI providing assistance or supporting role in healthcare equipment and devices may be a promising idea due to a lower obstacle of compatibility and connectivity.

In addition, AI providers could act as an assistant to help hospitals establish their own AI models and application systems using the accumulated healthcare big data. Since the healthcare industry has a geographical distribution, the AI model built on the hospital’s data would serve its patient group best. The model quality (e.g., the accuracy of prediction) could be good provided there is enough data in the electronic medical records (EMR).

It is necessary for providers to seize the opportunity to apply for patent protection and verification of equipment concerning healthcare AI products since too many R&D manufacturers, and relevant AI technologies are becoming increasingly simple without differences and advantages. The international community is aware of the rapid development of healthcare AI and has put forward relevant regulations and guidelines for reference, such as “The Software as Medical Device: Clinical Evaluation, Proposed Regulatory Framework for Modifications to Artificial Intelligence/Machine Learning-Based Software as a Medical Device” published by the US Food and Drug Administration (FDA) [21,22] and “The Medical Device of Artificial Intelligence/Machine Learning Technology: Technical Guidelines for Software Inspection and Registration” [23].

As for the target market, AI providers could sell their AI products to healthcare institutions with poor medical service quality (if the use of AI will improve the service quality), to countries with limited healthcare resources (if the domestic government will allow AI to partly replace healthcare workers and medical equipment), or to those with low medical insurance coverage (the cost of medical treatment is high, so people can use AI to properly assist in self-care management).

## 4. Critical Affecting Factors

Based on the above discussion, the critical factors affecting the deployment and scaling of healthcare AI are summarized below:

### 4.1. Policies and Regulations Amendment

The government should amend related policies and regulations to encourage the introduction of AI in health care, which could reduce or partially replace manpower. Moreover, the government has to subsidize substantively the development of AI in healthcare institutions to improve the overall healthcare quality and efficiency nationwide.

### 4.2. Top Executive Support

The introduction of AI into hospitals may require resource expenditure and continuous capital investment. Hence, hospital institutions should exert extra efforts to improve the knowledge of medical professionals on AI and its application even when short-term economic benefits may not seem apparent.

### 4.3. Clinical Actual Demand

Healthcare institutions should adopt/purchase AI products based on the actual demand of healthcare workers. Furthermore, AI needs to be integrated with existing processes and should not create an additional workload for medical workers.

### 4.4. User Department Consensus

Since AI is costly, it should not be used by only a few people. A consensus within the entire department should be established, putting forward the needs and expected benefits of AI for it to gain wide recognition and encourage healthcare workers’ participation in planning and introducing AI, and eventually, into using it regularly.

### 4.5. Dedicated AI Analysts

AI development is a complex process across all fields. It is necessary to set up special departments or units and prepare specific AI analysts to effectively and extensively promote the development of AI systems in various medical fields. If the AI analysts are only working part-time, or their work is designated to employees with other existing jobs and responsibilities (maybe statisticians or physicians), the progress may become slow, the subject may be limited, and their AI knowledge may be insufficient.

### 4.6. IS Department Supports

Whether it is the preparation of big data or the subsequent implementation of integration with HIS, it must be strongly supported by the IS department; otherwise, it will be difficult to complete. However, the IS department may already have a heavy routine and may only provide limited support on AI projects. Providing the IS department additional bonuses to encourage it to assist with AI projects during off-hours is a feasible approach.

### 4.7. Obvious Concrete Benefits

Since AI users and scientific studies have exaggerated AI’s functions, the introduction of AI should target clear aims and measurable benefits such as income increase, cost reduction, and improvement in quality and efficiency. Perceptive benefits such as user satisfaction and acceptance can be considered as well.

### 4.8. Improve AI’s Explainability

Studies have proven that AI performs excellently in prediction and is even better than that of healthcare workers. However, if AI cannot clearly explain the rules or basis of its prediction, the issue of AI being just a “black-box” will remain and continue to doubt the public, resulting in lower acceptance and use.

### 4.9. Continuous Optimization of Products

Along with environmental changes and technological progress, AI products require continuous optimization and improvement, such as model retraining, self-learning, and federated learning [24].

### 4.10. Easy to Install and Use

Since clinical work is already complex on its own, AI products should be easy to install and use and should work automatically as much as possible, but the final decision-making should ultimately remain within the healthcare workers. In addition, the AI should not interrupt or impede the clinical care process unnecessarily; otherwise, healthcare workers will avert from it.

### 4.11. Assistance rather than Replacement

The current AI still needs a lot of improvement. Although it can perform well in healthcare projects, it cannot match the overall judgment of an experienced medical professional. Medical decisions are based on numerous factors, including the emotional level of the patient, the stance of family members, and the socio-economic environment. Hence, hospitals should employ AI as an assistant rather than a replacement for healthcare workers.

### 4.12. Spontaneous rather than Compulsory

Regarding laws, practice, or even public perception, the use of AI still has a lot of problems, including ownership, accuracy, explainability, reliability, stability, morale affection, etc. Therefore, healthcare workers should have the right to choose whether or not to use AI rather than being imposed to do so.

## 5. Conclusions

The soft power of healthcare services and the hard power of the ICT industry in Taiwan have laid a strong foundation for developing healthcare AI. Although hospitals, technological manufacturers, and start-ups have launched enormous AI products, the mature economic scale and profit model remain unclear because large-scale cases of successful implementation of healthcare AI in medical institutions are still rare.

Based on the experience of the Chi Mei Hospital group that has deployed multiple AI applications, this research summarized the key influencing factors and possible responses that affected the development and diffusion of AI in medical institutions. This type of research is very important but less reported. We believe that relevant stakeholders or the so-called AI consumers, which include the government, medical institutions, end-users, and AI providers, should openly and fully cooperate to understand each other’s niches in AI development and jointly solve the problems in its development. Ideally, machines could be utilized to assist humans in generating higher quality predictions, with the final decisions and optimal actions being left to the latter [25]. This could realize AI-enabled hospitals with confidence.

Since this research is only based on Chi Mei Hospital’s view on the development of healthcare AI, it may not be enough to represent all hospitals. Chi Mei Hospital mainly develops AI applications based on its structured big data, which may not represent the experience of other types of healthcare AI (such as medical imaging). In addition, this study suggests that future researchers can explore the attitudes and expectations of more stakeholders thoroughly. Additionally, the differences between the development and introduction of AI in hospitals in different countries are worthy of comparative analysis.

## Data Availability

Not applicable.
